# Diagnosis of cervical lymphoma using a YOLO-v7-based model with transfer learning

**DOI:** 10.1038/s41598-024-61955-x

**Published:** 2024-05-14

**Authors:** Yuegui Wang, Caiyun Yang, Qiuting Yang, Rong Zhong, Kangjian Wang, Haolin Shen

**Affiliations:** Department of Ultrasound, Zhangzhou Affiliated Hospital to Fujian Medical University, No. 59 North Shengli Road, Zhangzhou, 363000 Fujian China

**Keywords:** Cancer, Computational biology and bioinformatics, Diseases

## Abstract

To investigate the ability of an auxiliary diagnostic model based on the YOLO-v7-based model in the classification of cervical lymphadenopathy images and compare its performance against qualitative visual evaluation by experienced radiologists. Three types of lymph nodes were sampled randomly but not uniformly. The dataset was randomly divided into for training, validation, and testing. The model was constructed with PyTorch. It was trained and weighting parameters were tuned on the validation set. Diagnostic performance was compared with that of the radiologists on the testing set. The mAP of the model was 96.4% at the 50% intersection-over-union threshold. The accuracy values of it were 0.962 for benign lymph nodes, 0.982 for lymphomas, and 0.960 for metastatic lymph nodes. The precision values of it were 0.928 for benign lymph nodes, 0.975 for lymphomas, and 0.927 for metastatic lymph nodes. The accuracy values of radiologists were 0.659 for benign lymph nodes, 0.836 for lymphomas, and 0.580 for metastatic lymph nodes. The precision values of radiologists were 0.478 for benign lymph nodes, 0.329 for lymphomas, and 0.596 for metastatic lymph nodes. The model effectively classifies lymphadenopathies from ultrasound images and outperforms qualitative visual evaluation by experienced radiologists in differential diagnosis.

## Introduction

Cervical lymphoma often presents as an abnormal enlargement of lymph nodes in the neck and must be differentiated from several other cervical lymphadenopathies (e.g., granulomatous inflammation, reactive inflammation, immune deficiency syndrome, tuberculosis, systemic lupus erythematosus, and metastasis) to develop an appropriate therapeutic plan^1,2^. The current diagnostic standard for cervical lymphadenopathy is a pathological examination, though views differ on the proper biopsy sample method for obtaining sufficient tissue for histologic examination of a given lymphadenopathy^3^. Excision biopsy is generally recommended for classification of lymphoma^4–6^, while only a needle biopsy is required for other cervical lymphadenopathies^7^. Excision biopsy has a greater risk of trauma-related symptoms and complications than needle biopsy^8^, so patients with enlarged cervical lymph nodes will usually be examined first with non-invasive imaging. If non-invasive imaging reveals benign lesions, patients can avoid the more invasive biopsy procedure.

Both computed tomography and ultrasound are commonly used for non-invasive diagnostic imaging of cervical lymphadenopathies^9,10^, though ultrasound is regarded as the first-line examination because it is radiation-free^11^. Lymph node diagnostic accuracy in differentiating benign from malignant lymph nodes has been improved by a new predictive scoring system based on ultrasound features^12^, while the diagnostic accuracy of lymphoma has improved with ultrasound-guided machine-learning models^13^. However, health professionals often encounter challenging cases with overlapping imaging features in differentiating metastasis from lymphoma only with conventional B-mode US and even with Doppler US^14^. It has been reported that a combination of ultrasound and contrast-enhanced ultrasound has good diagnostic value in distinguishing between cervical lymphadenitis and primary lymphoma^15^. However, these methods still depend on the subjectivity of the radiologist’s judgment in ultrasound interpretation. Besides, contrast-enhanced ultrasound is expensive and cumbersome to operate, which is not conducive to widespread implementation in grassroots hospitals.

Computer vision approaches to diagnostic image interpretation may overcome such limitations. Multiple computer vision techniques have enabled that convolutional neural network (CNN) can show good potential for the detection and classification of cervical lymphadenopathy^16,17^. Notably, CNNs have been demonstrated to be particularly suitable for computer vision, especially in image interpretation^18^. Representative CNN algorithms include Region-based Convolution Neural Networks (R-CNN), Fast R-CNN, Single Shot MultiBox Detector (SSD), and You Only Look Once (YOLO)^19–21^. YOLO is capable of identifying objects by localizing them with a bounding box and, at the same time, classifying them according to the probability to belong to a given class^22^. The YOLO series represents one-stage algorithms, which are more suited to practical applications than two-stage algorithms (such as Faster R-CNN) owing to their better balance between accuracy and speed^23^. Zhong et al.^24^ pointed that the YOLO model was superior to the Faster R-CNN model for the Helicobacter pylori detection task. YOLO-v7 leverages a trainable bag-of-freebies approach, enabling significant improvements in precision for real-time detection tasks without incurring additional inference costs. By integrating extend and compound scaling, it effectively reduce the number of parameters and calculations, resulting in a substantial acceleration of the detection rate^25^. To the best of our knowledge, no studies have applied this YOLO model to the diagnostic distinction of cervical lymphadenopathy.

In this study, we aimed to build an artificial intelligence diagnostic model based YOLO-v7 of cervical lymphadenopathy that would reduce subjective influence from radiologists and improve the accuracy of cervical lymphoma detection.

## Materials and methods

This retrospective study was approved by the research ethics committee of Zhangzhou Affiliated Hospital of Fujian Medical University (Protocol No. 2022KYB138). All experiments were performed in accordance with relevant guidelines and regulations. The need for informed consent was waived by the ethical committee with Zhangzhou Affiliated Hospital of Fujian Medical University.

### Dataset

Ultrasound images of cervical lymph nodes were collected from our hospital between January 2017 and June 2022 retrospectively, including B-mode and Doppler ultrasound. All lymph nodes had determinate pathological results. Patients with incomplete information and unclear pathological results were excluded. The entire dataset comprises three categories: benign lymph nodes (n = 2807), lymphomas (n = 1108), and metastatic lymph nodes (n = 4580).

Ultrasound images were captured with the Mindray Resona 7S Ultrasound Scanner (Mindray BioMedical, Shenzhen, China), Acuson S3000 Scanner (Siemens Medical Solutions USA, Malvern, PA), and Hitachi Vision Preirus Scanner (Hitachi Medical Corp., Chiba, Japan).

### Augmenting datasets, labeling images, and dividing image datasets

All images were resized to 640 × 640 pixels and then augmented via rotation and contrast changes to multiply and increase the sample size of the training dataset. Because the lymphoma sample set is relatively small, the amplification ratio of this subset is higher than that of the other two diseases. Augmentation variations for benign lymph node and metastatic lymph node datasets were: rotation (10° clockwise, 10° counter-clockwise, 80° clockwise, 80° counter-clockwise, 90° clockwise, 100° clockwise, 150° clockwise) and contrast change (by factor 0.5 and 1.5). Augmentation variations for the lymphoma dataset were: rotation (10° clockwise, 10° counter-clockwise, 45° clockwise, 45° counter-clockwise, 50° clockwise, 50° counter-clockwise, 80° clockwise, 80° counter-clockwise, 90° clockwise, 90° counter-clockwise, 100° clockwise, 100° counter-clockwise, 150° clockwise) and contrast change (by a factor of 0.5, 0.6, 1.0 and 1.5). After augmentation, the total dataset reached 93,814 images distributed as: benign lymph node (n = 28,070), lymphoma (19,944), and metastatic lymph node (n = 45,800) (Table 1). We utilized the “random.sample” function in Python to randomly divide the total dataset into three subsets: training, validation, and testing. The sampling process did not consider the lymph node types and randomly distributed the entire dataset. The training set received 90% of the images while the validation and testing sets each received 5%. The number of lymph nodes in each dataset is detailed in Table 2. The training set was used to train the model. The validation set was used to adjust the weight parameters of the model. The testing set was used to compare with the conducted qualitative visual evaluation by experienced radiologists.

**Table 1:** Number of images and enhancement rate for different types of lymph nodes.

	Original images	Amplification rate	Total images
Benign lymph nodes	2807	10	28,070
Lymphomas	1108	18	19,944
Metastatic lymph nodes	4580	10	45,800

**Table 2:** Cervical lymph node ultrasound images in different sets.

Dataset	Benign lymph node	Lymphoma	Metastatic lymph node	Total
Training	25,205	17,940	41,076	84,221
Validation	1388	988	2315	4691
Testing	1477	1016	2409	4902
Total	28,070	19,944	45,800	93,814

**Figure 1:**
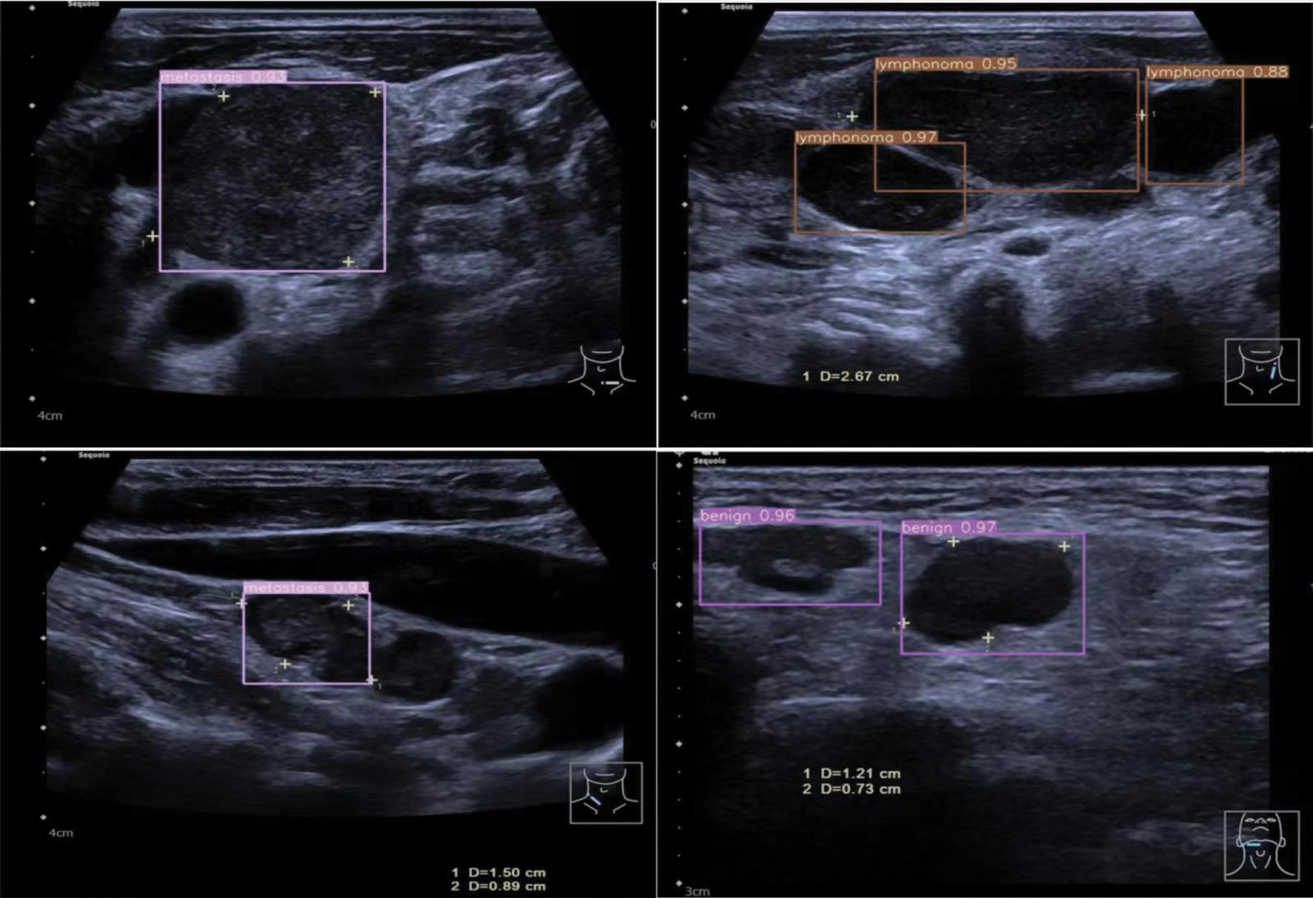
Sample images from each of the three classes with visual aids as to where the lymph nodes are. The lymph node was selected using LabelImg software by drawing a rectangular bounding box and assigning a category label such as “benign lymph node,” “lymphoma,” or “metastatic lymph nodes.” The result was saved in a .txt file.

An experienced radiologist (21 years) manually tagged images using the graphic marker software LabelImg. Using LabelImg software, the lymph node was selected with a rectangular bounding box and assign a category label to it, such as “benign lymph node”, “lymphoma” or “metastatic lymph nodes”. Then the result was saved into a .txt file (Fig. 1).

### Training YOLO-v7 model

We acquired both the source code document and the pre-trained YOLO-v7 weight model from GitHub. Originally trained on the MS COCO dataset from scratch by the primary authors^26^, we further trained the model using our own images. Throughout the training process, the model automatically fine-tuned its network structure and optimized its loss function.

Model training was performed on a machine with an Intel Core i9-12900H processor, 32 GB RAM, and a GPU with 8 GB memory. The hardware and software parameters of the training system are shown in Table 3.

**Table 3:** Hardware and software parameters of the training system.

Name	Parameters
Operating system	Windows 10, 64-bit
Processor	12th Gen Intel Core i9-12900H CPU 2.50 GHz
Installed RAM	32 GB
Graphics	NVIDIA, Intel Iris Xe
Graphics memory	8 GB
Development environment	Anaconda, PyCharm Community Edition 2022.2.3
Programming language	Python
Deep learning framework	PyTorch 1.11.6

**Figure 2:**
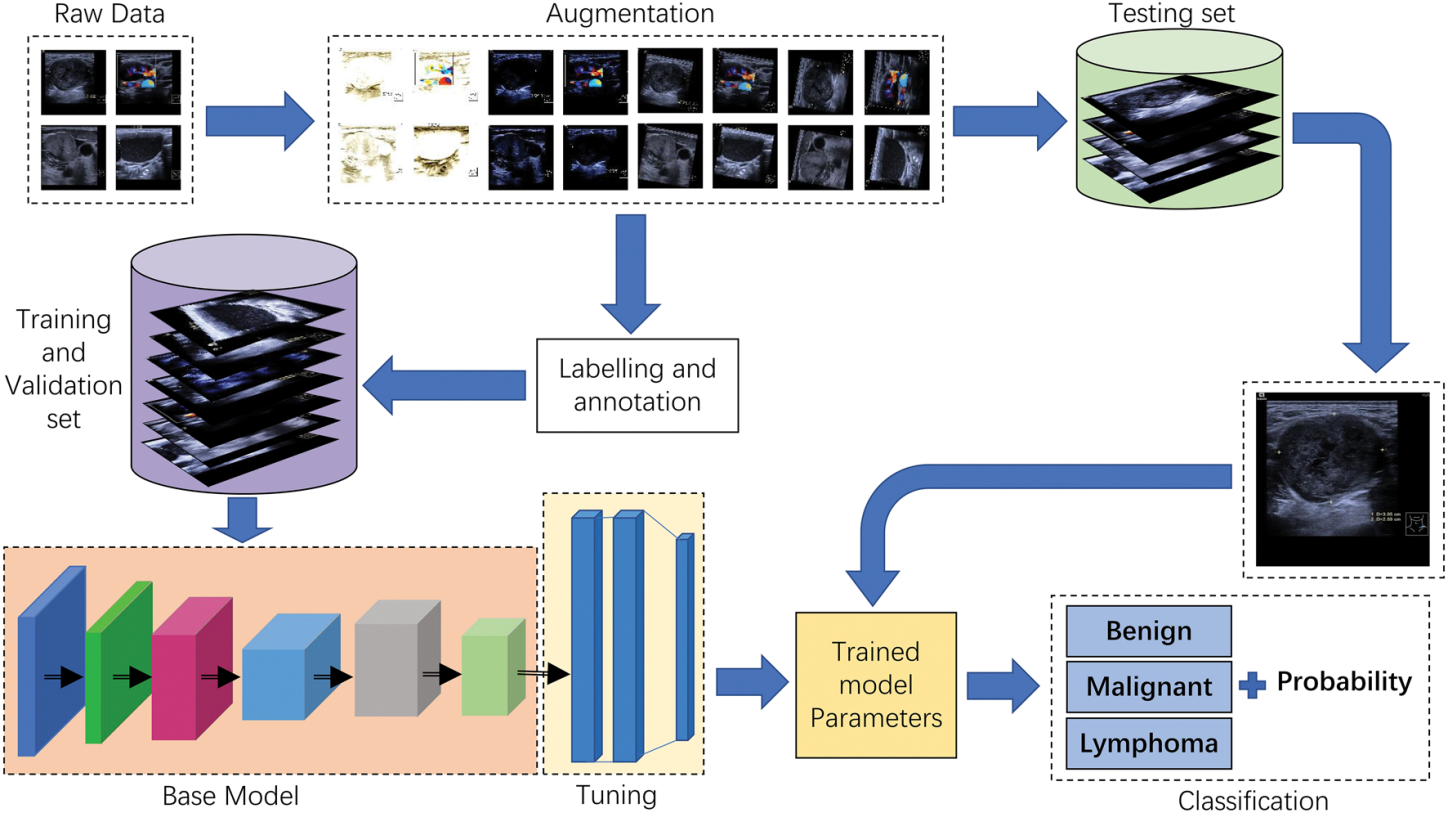
Flow chart of proposed YOLO-v7 model-based automatic cervical. lymphadenopathy detection. Collected images are resized to 640 × 640 pixels and enhanced by rotation and contrast. The dataset was randomly divided into 3 subsets for training, validation, and testing. Images from training and validation sets were marked using LabelImg software and the YOLO-v7 model was trained with these sets. Parameters were adjusted and the test images were submitted to the model to obtain the lymph node classifications and probabilities.

The YOLO-v7 model iterated 300 training epochs on the training set. The validation set was used to adjust weighting in the training model. The testing set was used to analyze the capability of the model. A block diagram of the complete methodology is shown in Fig. 2. The classification experiment was performed first on the validation subset and then on the testing subset, and the accuracy value, precision value, recall value, and F1 score of the model were automatically calculated by the software according to Eqs. (1)–(4). True Positive (TP) is a positive sample that is correctly classified. False Positive (FP) is a negative sample incorrectly classified as positive. True Negative (TN) is a negative sample that is correctly classified. False Negative (FN) is a positive sample incorrectly classified as negative. Background FN: The model misclassified the lesion into a background. Background FP: The model misclassified the background into a lesion. The mAP is used to measure the performance of the target detection algorithm. It is obtained from a comprehensive weighted average of the average accuracy of all categories detected. AP (average precision): For each category, calculate the area under its precision-recall curve to obtain AP. This represents the performance of the model at different levels of precision and recall. mAP (mean average precision): Take the average of all categories of AP to obtain mAP, which is a comprehensive evaluation of overall performance.

1 $$  \begin{gathered} {\text{Accuracy}} = \frac{{{\text{(TP}} + {\text{TN)}}}}{{\text{Total number of images}}} \hfill \\ \hfill \\ \end{gathered}  $$

2 $$  {\text{Precision}} = \frac{{{\text{TP}}}}{{\text{(TP + FP)}}}  $$

3 $$  {\text{Recall}}=\frac{{{\text{TP}}}}{{\text{(TP + FN)}}}  $$

4 $$  {\text{F}}1{\text{ Score}} = {2} \times \frac{{{\text{(Precison}} \times {\text{Recall)}}}}{{{\text{(Precison}} + {\text{Recall)}}}} $$

5 $$  AP = \int_0^1 {{\text{ p}}(r)dr}  $$

6 $$  {\text{mAP}} = \frac{{\sum\nolimits_{i = 1}^{\text{k}} {APi} }}{k} $$

### Visual evaluation by radiologists

**Figure 3:**
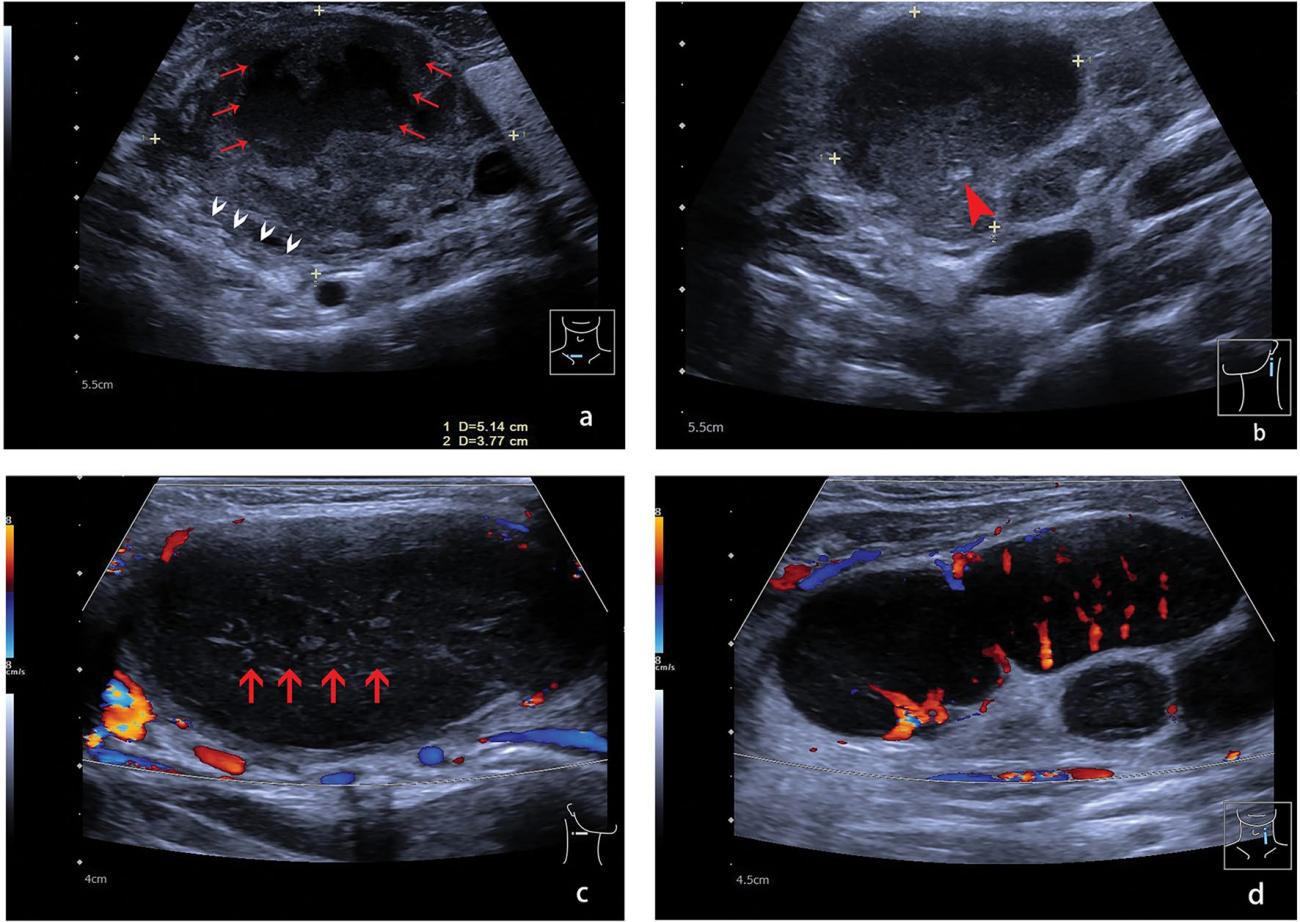
Ultrasound features of cervical lymph nodes. (**a**) Metastatic lymph node showing a long axis diameter (L) of 51.4 mm, short axis diameter (S) of 37.7 mm, L/S ratio < 2, absence of echogenic hilum, and a non-circumscribed margin (white arrows) with necrosis (red arrows); (**b**) Benign lymph node showing calcification (arrow); (**c**) Lymphoma showing grid-like echo (red arrows) and a peripheral vascular pattern; (**d**) Lymphoma showing a mixed vascular pattern.

The testing subset images were analyzed and diagnostically classified by two radiologists with more than 22 and 29 years of experience in ultrasonography. Radiologists were blinded to patient clinical information and pathological results. The ultrasound features of cervical lymphadenopathy are shown in Fig. 3 and include: absence of echogenic hilum, non-circumscribed margin, necrosis, calcification, grid-like echo, and peripheral vascular pattern^27^.

## Results

### Overall performance of YOLO-v7 model

**Figure 4:**
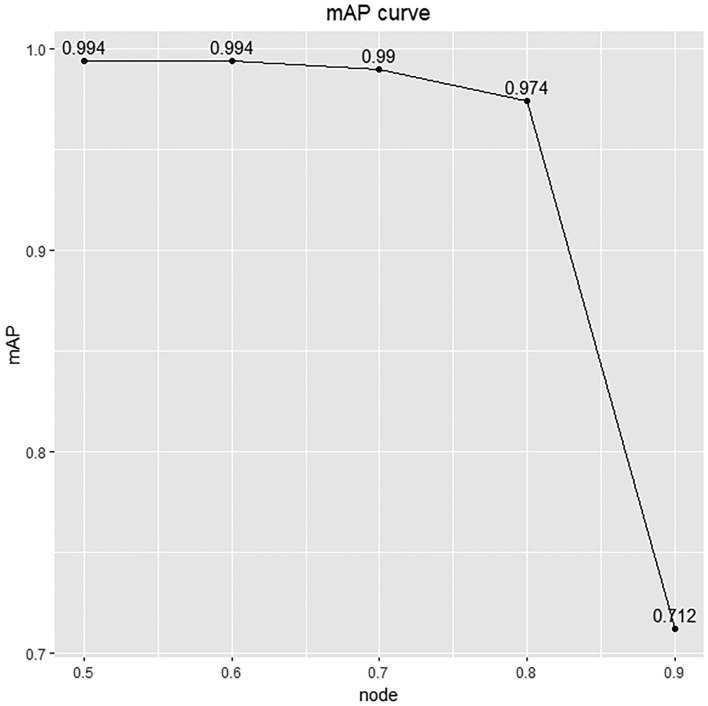
mAP value of YOLO-v7 model changed with different thresholds. The curve is a plot illustrating average precision values at various intersection-over-union thresholds. The x axis represents IoU thresholds, while the y axis represents the corresponding mAP. The model achieved the highest mAP at IoU thresholds of 50%.

**Figure 5:**
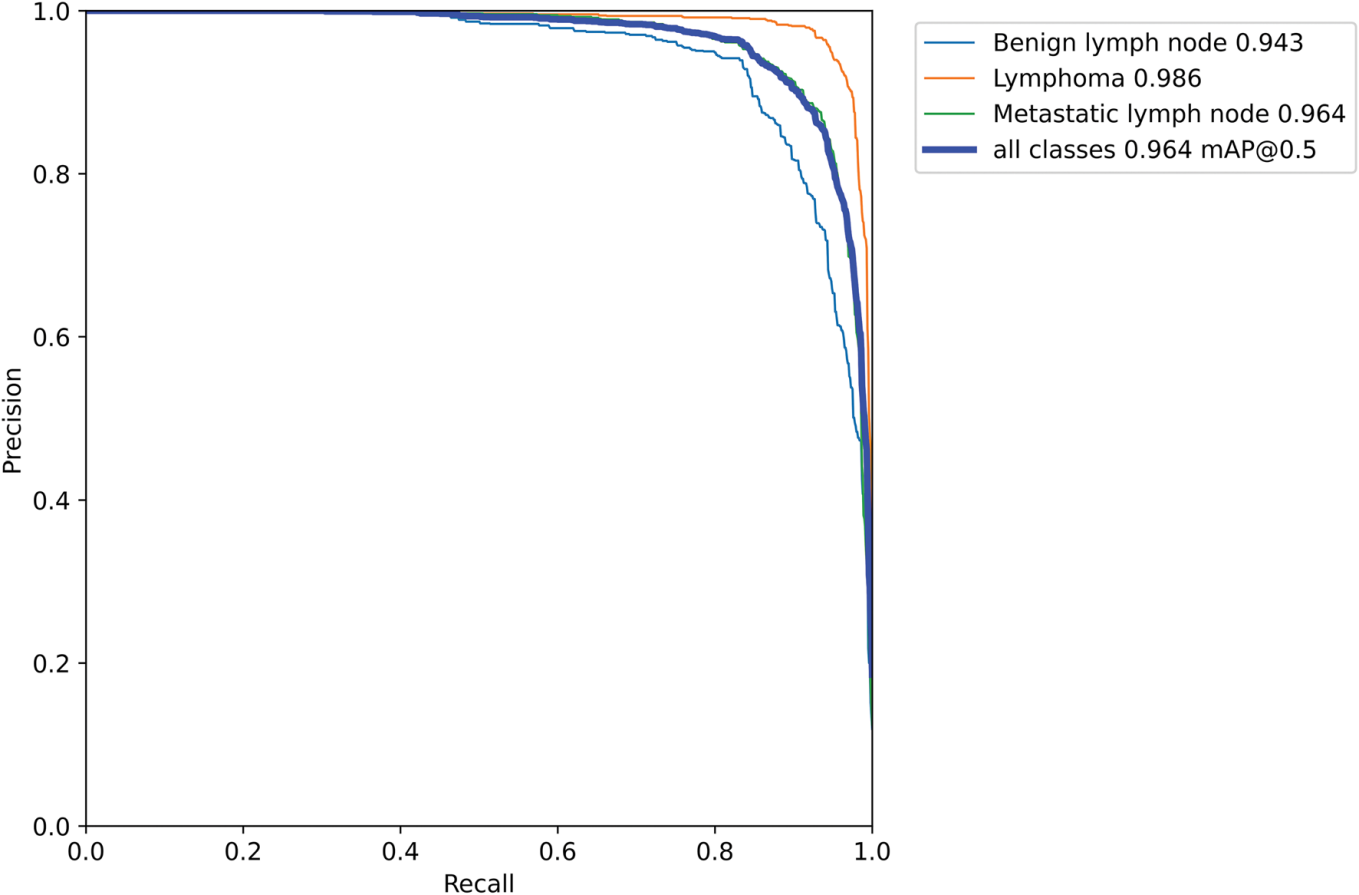
P–R curve of YOLO-v7 model. Precision is a measure of result relevance while recall is a measure of how many truly relevant results were returned. The average precision-recall values were 0.943, 0.986, and 0.964 for benign lymph node, lymphoma, and metastatic lymph node, respectively. Precision recall for all classes overall was 0.964.

After 200 iterations, the mAP, which is an indicator for measuring the quality of the detection model, gradually stabilizes. The model had the highest mAP at an intersection-over-union threshold of 50% (Fig. 4). At an intersection-over-union threshold of 50%, the model mAP on the testing dataset of 4691 images is 96.4% (Fig. 5).

**Figure 6:**
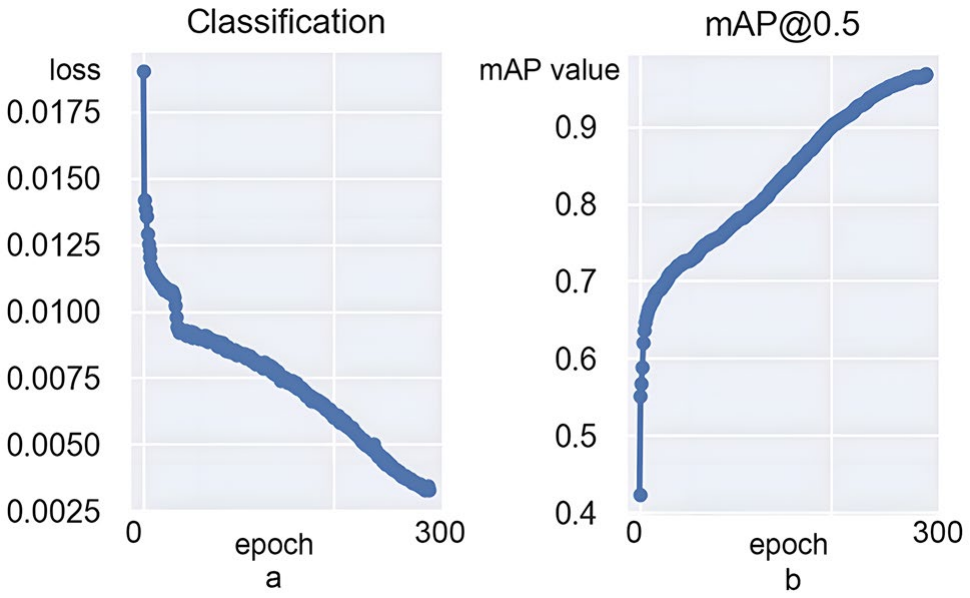
(**a**) Classification of loss. With increasing iterations, the YOLO-v7 algorithm curve gradually converges and the loss value of classification decreases. (**b**) The mAP is an indicator of the quality of the detection model. With increasing iterations, the mAP@0.5 also increases and the model quality improves and stabilizes.

**Figure 7:**
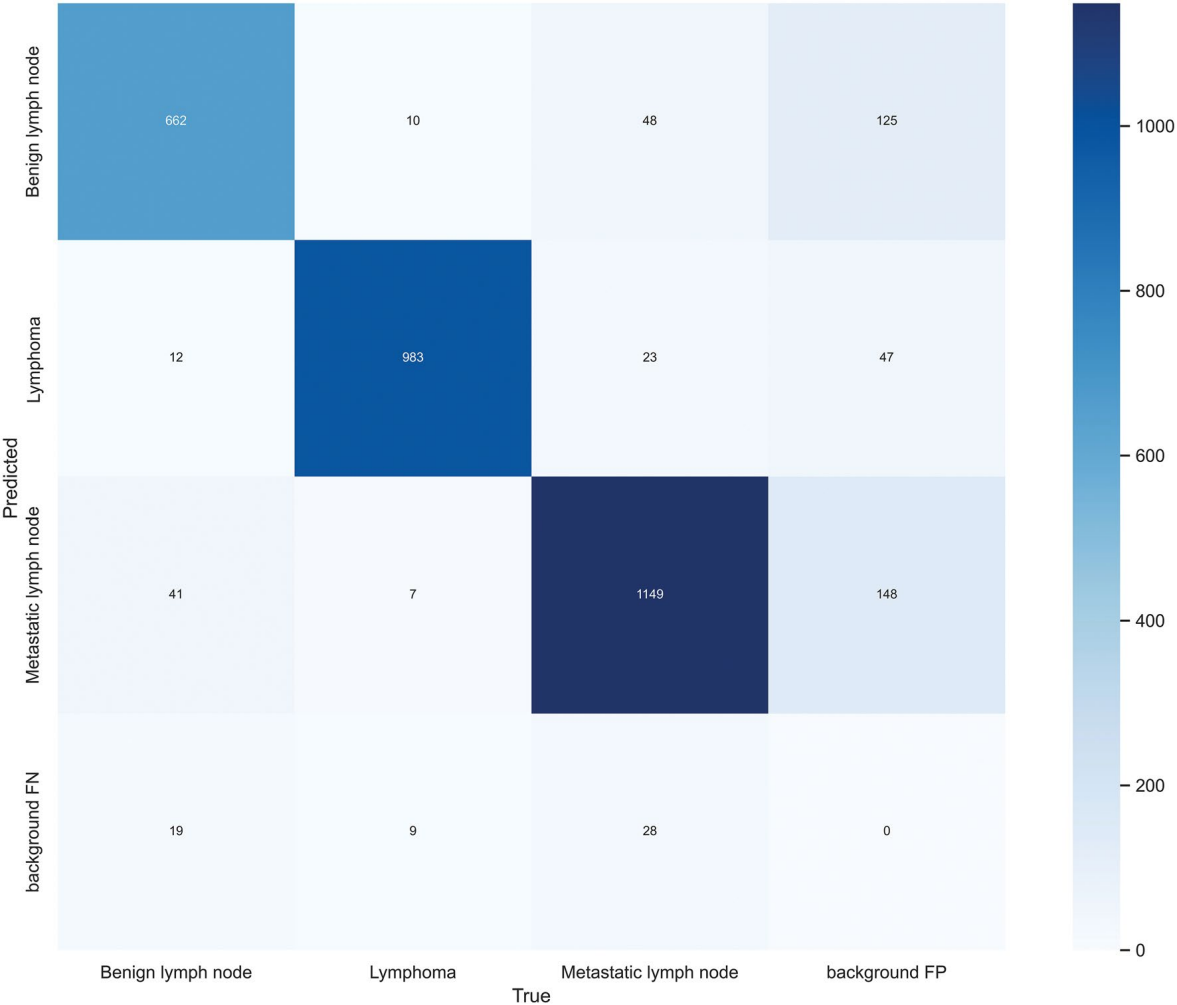
Confusion matrix for YOLO-v7 model. True Positive values were 662, 983, and 1149 for benign lymph nodes, lymphomas, and metastatic lymph nodes, respectively. *FP* False Positive, *FN* False Negative.

The loss function of the model (Fig. 6) shows that the YOLO-v7 algorithm curve gradually converges as the number of iterations increases and the loss value of classification decreases. After 300 iterations, the loss value of classification stabilizes near zero and the network essentially converges. The confusion matrix (Fig. 7) shows that the YOLO-v7 model has a recall value of 0.842, 0.925, and 0.882 for benign lymph nodes, lymphomas, and metastatic lymph nodes, respectively.

### Comparison of performance between the YOLO-v7 model and visual evaluation by radiologists

Table 4 shows the multi-class and individual class parameters for accuracy, recall, precision, and F1 score for the testing set. Visual evaluation by radiologists results were all lower. The recall value for lymphomas was only 0.237.

**Table 4:** Comparison of performance between YOLO-v7 model and qualitative visual evaluation by experienced radiologists.

Class	Accuracy	Precision	Recall	F1 score
YOLO-v7				
Benign	0.962	0.928	0.842	0.883
Lymphoma	0.982	0.975	0.925	0.949
Metastasis	0.960	0.927	0.882	0.904
Multi-class	0.952	0.944	0.883	0.912
QR				
Benign	0.659	0.478	0.408	0.440
Lymphoma	0.836	0.329	0.237	0.276
Metastasis	0.580	0.596	0.689	0.639
Multi-class	0.538	0.468	0.445	0.452

*QR* qualitative visual evaluation by experienced radiologists.

## Discussion

Ultrasound is the preferred diagnostic method for cervical lymphadenopathy. However, the diagnostic accuracy of ultrasound depends critically upon image quality, the professional experience of the radiologists, and the ultrasound instrument itself^28,29^. Object detector-based deep learning mode was used in detecting, segmenting, and classifying on lesions^30^. Before the emergence of YOLO, object detection algorithms such as DCNN generally required generating a large number of candidate regions and then classifying targets among them. Compared to region based methods, YOLO-v7 does not require early detection of potential target regions. It can output the category and show the location information of all targets by browsing the image only once. We trained a YOLO-v7 model that can identify the location of potential lesions on an entire ultrasound image and simultaneously classify lymph nodes as benign, lymphoma, or metastatic. The present study shows that the YOLO-v7 model is clearly superior to qualitative visual evaluation by experienced radiologists in a diagnostic test. The multi-class accuracy and F1 scores for the YOLO-v7 model were 0.952 and 0.912, respectively. And it indicated that the YOLO-v7 model can accurately identify lymphoma and also effectively distinguish benign and metastatic lymph nodes. To increase the amount of training data, the ultrasound images were augmented by rotation to train the model with a neural network having greater learning ability to discriminate features in a given image. A higher mAP indicates higher average detection accuracy and greater performance. Our model produced a maximum mAP of 96.4%, showing that the model can sensitively detect various classes of cervical lymphadenopathies, especially lymphomas; therefore, this model has achieved our goal. Compared with the YOLO-v7 model, the visual evaluation by experienced radiologists would miss most patients with lymphoma and cause unnecessary biopsies prior to lymph node resection.

In a study of ultrasound-based prenatal abnormality detection, the sensitivity range across different medical institutions was 27.5–96%^31^. This indicates that US is highly dependent on the experience and skills of the radiologists. So, it has become necessary to find a new technology that can overcome the subjectivity of US diagnosis. Our study showed that the precision value was only 0.329, indicating that many patients would be misdiagnosed with lymphoma and undergo unnecessary lymphadenectomies. This further indicates that visual evaluation by radiologists depends greatly on the personal experience of the radiologist and is therefore highly subjective. It remains difficult to accurately differentiate lymph node diseases, even for senior radiologists. To address these limitations, there were some studies combined conventional ultrasound, shear-wave elastography, and contrast-enhanced ultrasonography to detect the stiffness, perfusion pattern, and characteristics of lymph nodes. Experiments have shown that multimodal ultrasonography is a valuable tool for differentiating between benign and malignant lymphadenopathies^32^. Some advanced automated ultrasound image analysis methods have been also developed to improve the objectivity, accuracy, and intelligence of ultrasound diagnostics and image-guided intervention^33,34^. The most widely used method is an automated feature extraction and informatics analysis using radiomics, but the image segmentation step usually relies on manual delineation that introduces errors in image feature calculation^33^. This paper presents an automated and accurate deep-learning-based cervical lymphadenopathy diagnostic technique that does not involve extra feature extraction operations. It is convenient and highly accurate. The accuracy, precision, recall and F1 score of the three pathological types of lymph nodes of the model are all better than the qualitative visual evaluation of experienced radiologists. Given this accuracy and stability, we believe that the YOLO-v7 model is superior to visual evaluation by radiologists for the differential diagnosis of lymphadenopathy.

In clinical practice, it's common for US images to feature calipers and body markers. Consequently, these elements were retained in the US images utilized to train the YOLOv7 model. While this decision might introduce some interference during the training phase, it enhances the model's ability to accurately identify target objects and minimize errors caused by non-target elements in subsequent applications.

The main limitation of this project is the scope; the study is retrospective and performed at a single institution. Prospective multicenter studies will be conducted in the future to further validate our findings. In addition, our study only focuses on ultrasound diagnostics. In future research, we will collect more data from computed tomography and magnetic resonance imaging to further train a more efficient YOLOv7 model.

In conclusion, The YOLO-v7 model classifies ultrasound images effectively and outperforms doctor’s recognition in the differential diagnosis of cervical lymphadenopathy. This suggests that the YOLO-v7 model has high clinical applicability and could be used for rapid screening at low cost.

## Data Availability

The datasets used and analysed during the current study available from the corresponding author on reasonable request.

## Code availability

The source code of YOLO-v7 model was publicly obtained from Github. The project code has been uploaded to Github. (https://github.com/YCY2023/yolo-v7-code.git).
